# Type 2 diabetes mellitus facilitates *status epilepticus* in adult rats: Seizure severity, neurodegeneration, and oxidative stress

**DOI:** 10.1002/epi4.12905

**Published:** 2024-02-06

**Authors:** Karen Paola Ramos‐Riera, Luis Beltrán‐Parrazal, Consuelo Morgado‐Valle, Francisca Pérez‐Severiano, Pablo Eliasib Martínez‐Gopar, María Leonor López‐Meraz

**Affiliations:** ^1^ Doctorado en Investigaciones Cerebrelaes Universidad Veracruzana Xalapa Mexico; ^2^ Instituto de Investigaciones Cerebrales Universidad Veracruzana Xalapa Mexico; ^3^ Laboratorio de Neurofarmacología Molecular y Nanotecnología Instituto Nacional de Neurología y Neurocirugía “Manuel Velasco Suárez” CDMX Mexico

**Keywords:** hyperglycemia, oxidative stress, seizures, status epilepticus, streptozocin

## Abstract

**Objective:**

The goal of this research was to evaluate the effect of DM type 2 (DM2) on SE severity, neurodegeneration, and brain oxidative stress (OS) secondary to seizures.

**Methods:**

DM2 was induced in postnatal day (P) 3 male rat pups by injecting streptozocin (STZ) 100 mg/kg; control rats were injected with citrate buffer as vehicle. At P90, SE was induced by the lithium–pilocarpine administration and seizure latency, frequency, and severity were evaluated. Neurodegeneration was assessed 24 h after SE by Fluoro‐Jade B (F‐JB) staining, whereas OS was estimated by measuring lipid peroxidation and reactive oxygen species (ROS).

**Results:**

DM2 rats showed an increase in latency to the first generalized seizure and SE onset, had a higher number and a longer duration of seizures, and displayed a larger neurodegeneration in the hippocampus (CA3, CA1, dentate gyrus, and hilus), the piriform cortex, the dorsomedial nucleus of the thalamus and the cortical amygdala. Our results also show that only SE, neither DM2 nor the combination of DM2 with SE, caused the increase in ROS and brain lipid peroxidation.

**Significance:**

DM2 causes higher seizure severity and neurodegeneration but did not exacerbate SE‐induced OS under these conditions.

**Plain Language Summary:**

Our research performed in animal models suggests that type 2 diabetes mellitus (DM2) may be a risk factor for causing higher seizure severity and seizure‐induced neuron cell death. However, even when long‐term seizures promote an imbalance between brain pro‐oxidants and antioxidants, DM2 does not exacerbate that disproportion.

**Key points**

- The chronic elevation of glucose in type 2 diabetes (DM2) may contribute to increased neurodegeneration when a severe insult like *status epilepticus* (SE) occurs.
- Our study showed that DM2 exacerbates SE severity and neurodegeneration.
- Our findings indicate that SE elicits greater brain oxidative damage than DM2 hyperglycemia alone.
- Our results demonstrate that DM2 exacerbates SE severity and neurodegeneration without affecting seizure‐induced lipid peroxidation or reactive oxygen species.

## 1 INTRODUCTION

Epilepsy has multiple causes including structural abnormalities, genetic factors, infections, and metabolic changes.^1^ *Status epilepticus* (SE) is a neurological emergency consisting of a prolonged, non‐stopping seizure. It is associated with a high mortality and may lead to widespread neuronal death and network changes in several brain regions including the limbic areas.^2^

Uncontrolled hyperglycemia can cause severe health alterations such as cardiovascular diseases and central nervous system dysfunctions. Clinical studies have shown comorbidity between diabetes mellitus (DM) and epilepsy,^3^, ^4^ and recently, epilepsy is associated with a greater prevalence of type 2 diabetes mellitus (DM2), the most common type of DM around the world.^5^ Around 19%–25% of DM2 patients have reported seizures, including a higher incidence of SE.^6^ However, experimental evidence about this relationship is limited. Mice with type 1 DM (DM1) have longer kainate‐induced seizures,^7^ whereas rats with DM1 manifest a higher percentage of severe seizures after lithium‐pilocarpine application.^8^

On the other hand, SE promotes an excessive production of free radicals and reactive oxygen species (ROS), which in turn facilitates the establishment of oxidative stress (OS) and contributes to neurodegeneration and epileptogenesis.^9^, ^10^, ^11^ DM also increases ROS generation by affecting different metabolic pathways including glycolysis, glucose auto‐oxidation, sorbitol pathway, protein glycation, and mitochondrial electron transport chain activity,^12^ and decreases the body's endogenous antioxidant response,^13^ which generates a redox imbalance and triggers OS. This redox imbalance may contribute to DM‐associated brain complications like seizures.

Despite the previous evidence, the association between DM2 and epilepsy has not been deeply explored; moreover, the mechanisms that facilitate seizures in the presence of DM are not fully understood. Therefore, this study aimed to characterize SE susceptibility in male Wistar rats with DM2 by analyzing the seizure severity, the neurodegeneration, and the OS in several brain regions.

## 2 METHODS

### 2.1 Experimental subjects

All animal procedures were performed following the technical specifications for the production, care, and use of laboratory animals from Mexico (NOM‐062‐ZOO‐1999) and the Guidelines for Care and the Use of Experimental Animals the Guide for the Care and Use of Laboratory Animals of the National Institutes of Health and under the ARRIVE guidelines. The Internal Committee for the Care and Use of Laboratory Animals of Instituto de Investigaciones Cerebrales, Universidad Veracruzana approved the experiments for this study (CICUAL‐IICE 2018–007).

Wistar rats were bred in our local colony (Instituto de Investigaciones Cerebrales, Universidad Veracruzana, Mexico) and kept in environmental conditions of temperature (22–25°C) and relative humidity (65%–75%), with 12 h light–dark cycles (lights on at 07:00 h) and free access to drinking water and standard food for rodents (Labdiet). The day of birth of the rats was considered as postnatal day 0 (P0). Sex was assessed on P3 and pups were kept with their mothers until P21 when they were weaned. Posteriorly, male rats were housed in collective acrylic boxes (43 × 53 × 20 cm) in groups of 5–6 individuals.

### 2.2 Experimental groups

Three‐day‐old rat pups were divided into two groups: The control group (CTRL) received the vehicle (VEH) for streptozocin (STZ), and the DM2 group received a single STZ dose. Subsequently, to evaluate the effect of DM2 on SE in adulthood, rats were divided in 4 experimental groups: (1) the first group was administered with VEH at P3 but were not exposed to SE at P90 (CTRL‐CTRL), (2) the second group was administered with STZ at P3 but were not exposed to SE at P90 (STZ‐CTRL), (3) the third group was administered with VEH at P3 and exposed to SE at P90 (CTRL‐SE), and (4) the fourth group was administered with STZ at P3 and exposed to SE at P90 (STZ‐SE) (Figure S1).

### 2.3 Type 2 diabetes model

On day P3, a group of rats were injected with 100 mg/kg s.c. of STZ (Sigma) dissolved in 0.1 M pH 4.5 citrate buffer as vehicle (Citric acid, J. Baker). The vehicle (VEH, 2 mL/kg body weight) was administered to control rats (CTRL).^14^, ^15^ Only the rats that presented glycemia ≥200 mg/dL were included in the study.

All rats, CTRL and STZ, had a blood sample taken from the lateral tail vein on days P30, P60, and P90 to monitor their serum glucose levels. Before sample extraction, the tail skin was cleaned with 70% ethanol, and a drop of blood was obtained by puncturing the lateral tail vein with a sterile lancet. Glucose levels were tested with a OneTouch UltraMini glucometer. Additionally, the body weight of rats was recorded on days P30, P60, and P90.

### 2.4 Determination of biochemical indicators of diabetes

A group of rats neonatally administered with STZ or vehicle (CTRL group) were euthanized by decapitation at P90, their brain was used for OS determination as described below, and 2–3 mL of blood from the carotid artery was collected in tubes with or without anticoagulant (EDTA) to evaluate their metabolic status. Blood samples were stored at 4°C until analysis. Serum was obtained immediately after clot retraction and centrifugation at 3000 rpm for 10 min and stored at −18°C until analysis. The biochemical indicators of diabetes analyzed were glycated hemoglobin, urea and blood urea nitrogen, creatinine.

Urinary biochemical indicators of diabetes were also analyzed. For this purpose, urine from CTRL and STZ rats was collected for 1 h previous to euthanasia; access to water and food was restricted during this period. We determined protein, glucose, ketone bodies, and specific gravity by using urinalysis reagent strip pads (Mission). Once the urine was obtained, strip pads were impregnated with the samples, and the results were compared with the color chart found on the tube labeling for each parameter. The results were obtained by direct comparison with the color chart (Supplementary material and methods).

### 2.5 Induction of *status epilepticus*

At P89 rats were i.p. injected with lithium chloride (3 mEq/kg, Sigma), and 18 h later, SE was induced by subcutaneous injection of pilocarpine hydrochloride (30 mg/kg, Sigma).^16^ Seizure severity was evaluated with the behavioral scale described by Racine.^17^ SE was defined as continuous seizure activity for at least 30 min. 1 h after SE onset, rats received an injection of 10 mg/kg i.p. of diazepam (Relazepam Vet, PiSA) to stop SE and to increase survival (Supplementary material and methods).

### 2.6 Determination of neurodegeneration

#### 2.6.1 Transcardiac perfusion and tissue processing

Twenty‐four hours after SE, a group of rats were anesthetized with 120 mg/kg, i.p. sodium pentobarbital (Cheminova) and transcardially perfused with 4% paraformaldehyde (dissolved in a 0.1 M phosphate solution, pH 7.4). The brains were embedded in paraffin blocks and serial 10 μm coronal sections were obtained at the level of the dorsal hippocampus (3.36 mm posterior to bregma^18^) (Supplementary material and methods).

#### 2.6.2 Fluoro‐Jade B (F‐JB) stain

We assessed neurodegeneration by using the F‐JB (Chemicon International) staining (Supplementary material and methods). The brain regions evaluated were the granular layer of the dentate gyrus (DG), the hilus, CA3, CA2, and CA1 pyramidal layer of the hippocampus, layer II of the piriform cortex, the dorsomedial nucleus of the thalamus (which includes the medial dorsal medial, central, and lateral nuclei), the lateral amygdala (including the lateral dorsolateral, lateral ventromedial, and lateral ventrolateral nuclei), the medial amygdala (including the medial posterodorsal nucleus and medial posteroventral nucleus), and the cortical amygdala (including the posteromedial cortical nucleus and area amygdala anterolateral hippocampus). The counts are reported as the number of cells/mm^2^ for each brain region evaluated.

### 2.7 Oxidative stress analysis

To evaluate the OS, we measured the products of lipid peroxidation (LP) and the formation of ROS 6 and 24 h after SE. The rats were decapitated, and the brain regions of interest (hippocampus, amygdala‐piriform cortex region, and thalamus) were quickly dissected on ice. The tissue samples were kept deep‐frozen at −80°C until analysis. (Supplementary material and methods).

### 2.8 Lipid peroxidation

The brain tissue was homogenized in saline, and one aliquot was mixed with a chloroform–methanol solution (2:1 vol/vol). The chloroform phase was analyzed with a fluorescence spectrophotometer (Perkin Elmer LS50B) using 370 nm and 430 nm excitation and emission wavelengths, respectively. Results are expressed as F.U./mg protein.^19^

### 2.9 Reactive oxygen species

Samples were incubated with TRIS‐ HEPES buffer (18:1) and 2′,7′‐dichlorodihydrofluorescein diacetate (50 μM) at 37°C for 1 h. The fluorescence of each sample was determined on an FLx800 multi‐plate reader (Biotek Instruments, Inc.), using 488 nm and 525 nm as excitation and emission wavelengths, respectively. Results are expressed as pmol DCF/mg protein/60 min.^20^

### 2.10 Statistical analysis

The Shapiro–Wilk test was used to assess the normal distribution of data. Blood glucose levels and body weight were analyzed with a two‐way analysis of variance (ANOVA) for repeated samples, considering as factors: (1) neonatal treatment (VEH and STZ) and (2) postnatal time course (30, 60, and 90); when necessary, a Bonferroni post hoc test was used. Biochemical indicators of diabetes in blood (HbA1c, urea, BUN, creatinine) and urine (protein, glucose, and specific gravity) did not show a normal distribution, and differences between groups were analyzed with the Mann–Whitney *U*‐test for unpaired samples. Seizure severity data (latency to the first generalized seizure and the SE, and the number and duration of generalized seizures) did not show a normal distribution. Thus, differences between groups were assessed by a Mann–Whitney *U*‐test for unpaired samples. Similarly, neurodegeneration was analyzed with a Mann–Whitney *U*‐test for unpaired samples. Data from ROS and LP analysis (6 and 24 h) were analyzed by a two‐way analysis of variance, considering as factors (1) neonatal treatment (VEH and STZ) and (2) SE induction (CTRL and SE); when necessary, a Bonferroni post hoc test was performed. To identify a relationship between glycemia levels and brain LP or ROS, a Pearson's correlation was performed. For all comparisons, a significance level of *p* < 0.05 was considered, and Prisma GraphPad v10.2 was used.

## 3 RESULTS

### 3.1 Development of type 2 diabetes

STZ rats showed higher blood glucose levels and lower body weight when compared to controls. The ANOVA analysis showed that both the neonatal STZ treatment (*p* < 0.001) and the postnatal time course (*p* < 0.001) were significant factors influencing glucose level, and a significant interaction between both factors was found (*p* < 0.001). The Bonferroni test showed an increase in blood glucose levels between P60 and P90 in STZ rats (*p* < 0.05); however, at P30, there was no difference in glucose concentration between the CTRL and STZ groups. In the CTRL group, the serum glucose concentration remained constant throughout the evaluation (Figure S2). In addition, the ANOVA analysis showed that both the neonatal treatment (*p* < 0.001) and the postnatal time course (*p* < 0.001) were significant factors influencing body weight. The interaction between these two factors also showed statistically significant differences (*p* = 0.001). When performing the multiple comparison test, results showed that the body weights of the CTRL and STZ rats were similar at P30, but this parameter was lower in STZ rats than in CTRL rats at P60 and P90 (Figure S2).

Levels of HbA1c were determined in CTRL and STZ rats to monitor the glucose course over 120 days. Analysis showed that STZ‐administered rats had a significant increase in HbA1c levels when compared to CTRL rats [(*p* = 0.001); Median 4.4 and 3.7, respectively]. In addition, biochemical indicators of kidney function in blood were determined. Urea (*p* = 0.009), BUN (*p* = 0.022), and creatinine (*p* = 0.007) levels were significantly higher in rats with STZ in comparison with CTRL rats. Parameters indicative of renal function showed that the urine of the STZ rats was denser than the urine from the CTRL rats (*p* = 0.001). In addition, STZ rats showed the presence of protein in urine (*p* = 0.004), which was not detected in the urine from the CTRL group rats. None of the control rats had glucose in their urine; however, 21% of the rats administered with STZ showed the presence of glucose in the urine, whose levels ranged between 1000 and 2000 mg/dL. Finally, ketone bodies were not detected in any of the experimental groups (Table S1).

Altogether, these results suggest that rats at P90 show biochemical changes indicative of initial DM2 following postnatal STZ injection.

### 3.2 *Status epilepticus* severity

**FIGURE 1:**
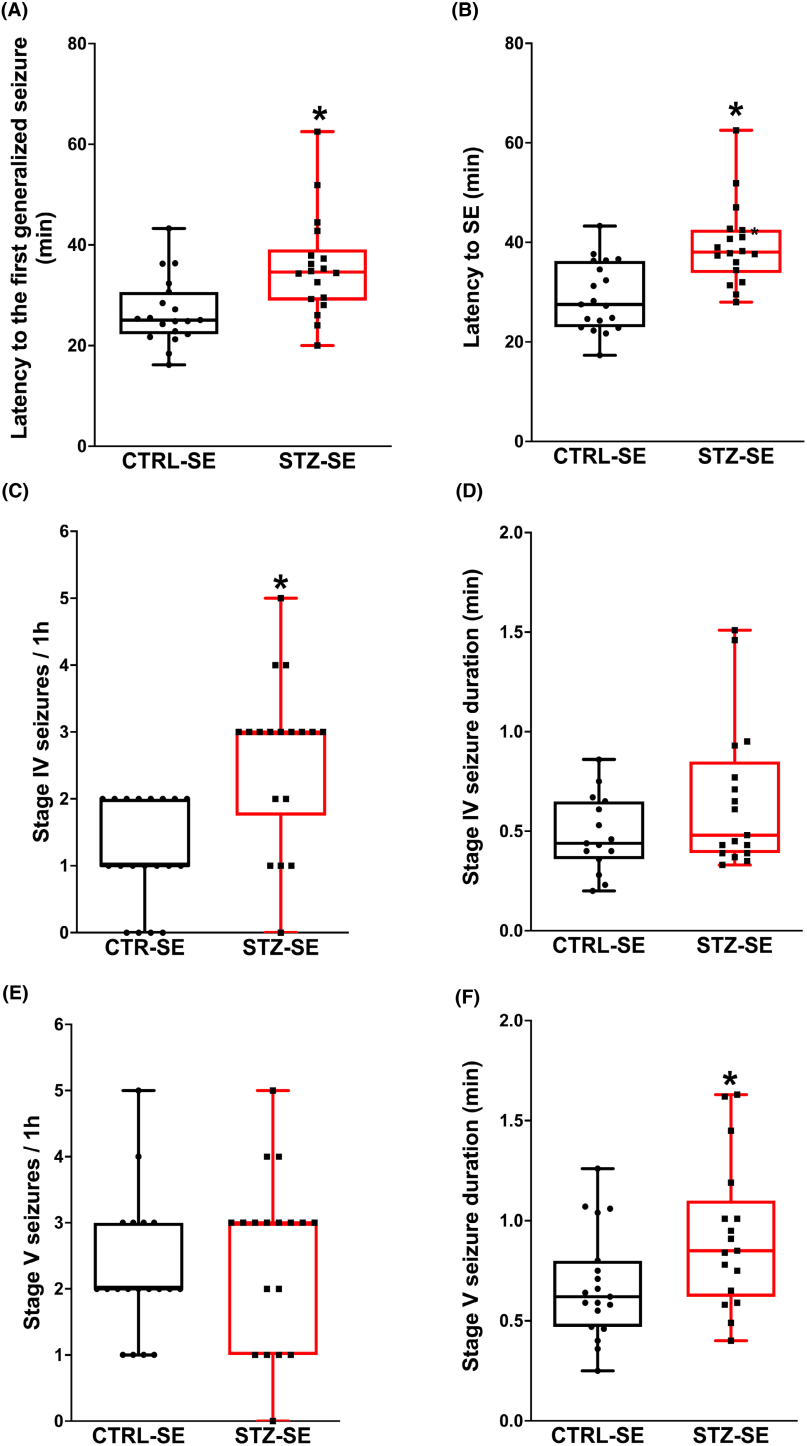
Seizure severity in rats treated with streptozocin (STZ‐SE) or vehicle (CTRL‐SE). (A) Latency to the first generalized seizure, (B) latency to *status epilepticus* (SE), (C) number and (D) duration of stage IV generalized seizures, (E) number, and (F) duration of stage V generalized seizures. Data were analyzed with the Mann–Whitney *U*‐test and are expressed as the median and interquartile range; the whiskers correspond to the minimum and maximum values for each group. Individual values for each rat are plotted in the graphs. CTRL‐SE Group *n* = 19, STZ‐SE Group *n* = 18. *p < 0.05.

STZ‐SE rats showed increased latency to the first generalized seizure (*p* = 0.002) and to the SE onset (*p* = 0.002) in comparison with the CTRL‐SE group. STZ‐SE rats had more generalized seizures (stage IV or V) compared with CTRL‐SE (*p* < 0.001), and specifically STZ‐SE rats presented a higher number of stage IV seizures than CTRL‐SE rats (*p* < 0.001); however, theirs durations were similar in both groups (*p* = 0.27). In addition, no difference in number of stage V seizures was found between STZ‐SE and CTRL‐SE rats (*p* = 0.39), but the duration of these seizures was longer in STZ‐SE rats when compared to CTRL‐SE rats (*p* = 0.04) (Figure 1).

### 3.3 Neurodegeneration after seizures

**FIGURE 2:**
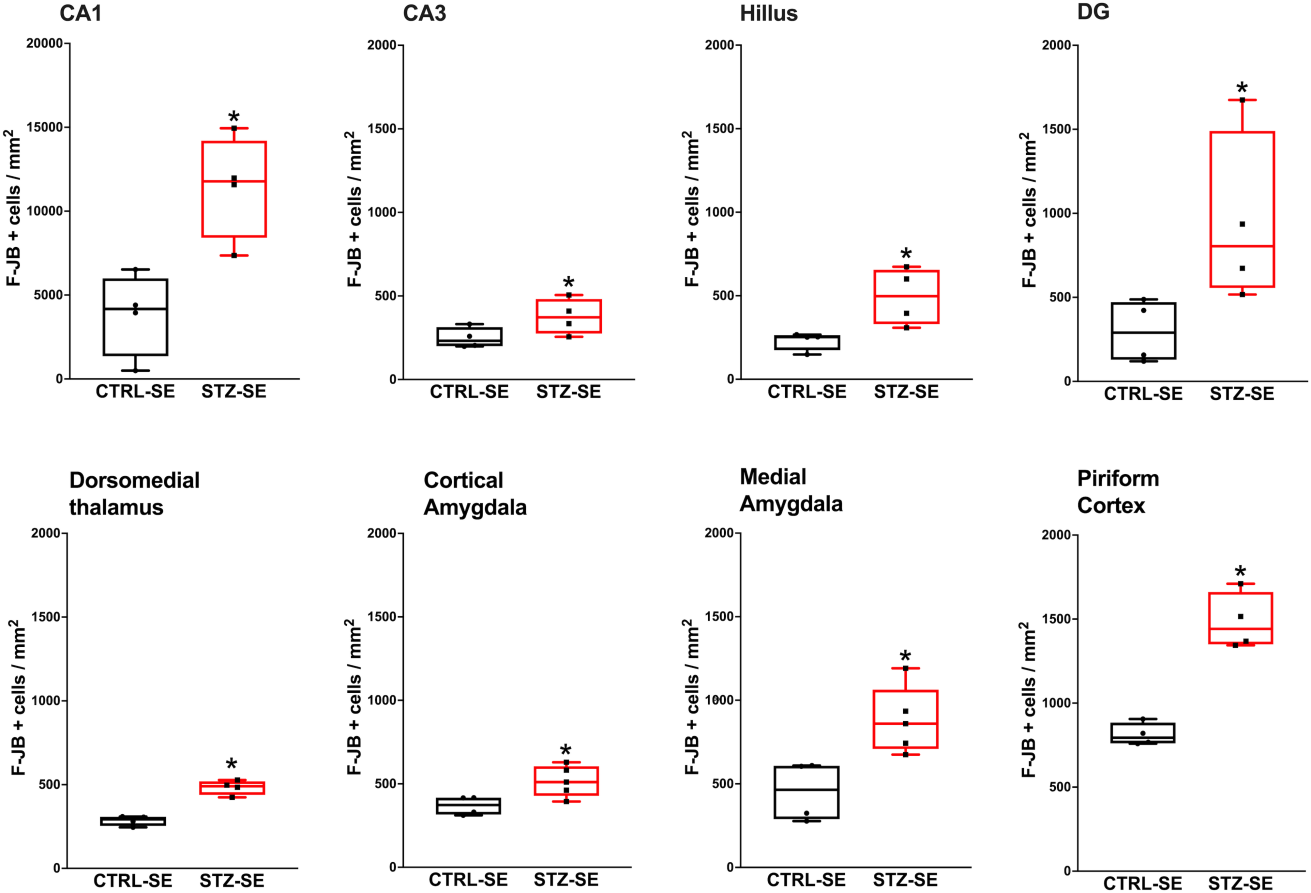
Neuronal neurodegeneration in rats neonatally treated with streptozocin (STZ‐SE) or vehicle (CTRL‐SE) and induced to *status epilepticus* (SE). Number of positive Fluoro‐Jade B (F‐JB) cells in the hippocampus, pyriform cortex, thalamus, and amygdala. Data were analyzed with the Mann–Whitney *U*‐test and are expressed as the median and interquartile range; the whiskers correspond to the minimum and maximum values for each group. Individual values for each rat are plotted in the graphs. CTRL‐SE Group *n* = 4, STZ‐SE Group *n* = 4. *p < 0.05.

No degenerative cells were detected in CTRL‐CTRL and STZ‐CTRL rats. In contrast, STZ‐SE rats presented a higher number of F‐JB positive cells in the DG (*p* = 0.02), the hilus (*p* = 0.02), CA3 (*p* = 0.02), and CA1 (*p* = 0.02) fields when compared to CTRL‐SE rats. No significant difference between both experimental groups was observed in the hippocampal CA2 area (*p* = 0.22). An increased number of F‐JB positive cells was also detected in the piriform cortex (*p* = 0.02), the dorsomedial nucleus of the thalamus (*p* = 0.028), and the medial (*p* = 0.01) and cortical nuclei (*p* = 0.01) of the amygdala from STZ‐SE rats when compared to CTRL‐SE rats. No significant differences were found between the two experimental groups regarding the number of F‐JB positive cells in the lateral amygdala nucleus (*p* = 0.48) (Figure 2).

### 3.4 Oxidative stress after seizures

#### 3.4.1 Lipid peroxidation

**FIGURE 3:**
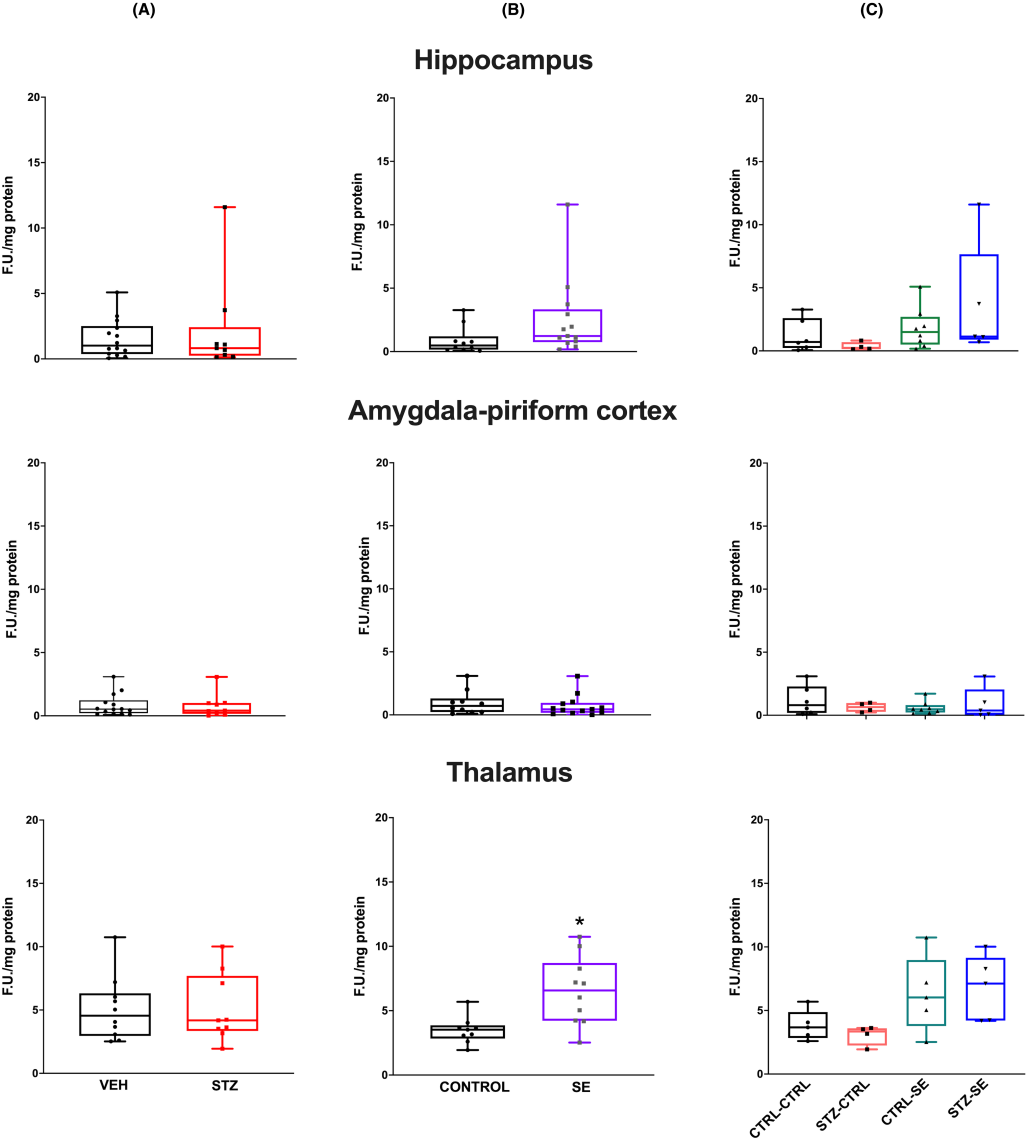
Lipid peroxidation (LP) levels 6 h after SE in the hippocampus, the amygdala–pyriform regions, and the thalamus in the different experimental groups. Figures show (A) the effect of neonatal treatment (vehicle, VEH or streptozocin, STZ), (B) the effect of *status epilepticus* (SE vs no seizures, CTRL), and (C) their interaction. Data were analyzed using a two‐way ANOVA and are expressed as the median and interquartile range; the whiskers correspond to the minimum and maximum values for each group. Individual values for each rat are plotted in the graphs CTRL‐CTRL *n* = 5, STZ‐CTRL *n* = 4, CTRL‐SE *n* = 5, STZ‐SE *n* = 7. *p < 0.05.

LP was evaluated 6 and 24 h after SE under the different experimental conditions and analyzed with a two‐way ANOVA. At 6 h, neither the neonatal STZ treatment (*p* = 0.64), nor the induction of SE (*p* = 0.08) nor the interaction between both factors (*p* = 0.20) were significant factors in LP changes in the hippocampus. Similarly, neither the neonatal STZ treatment (*p* = 0.78), nor the induction of SE (*p* = 0.71) nor the interaction between both factors (*p* = 0.28) significantly affected LP levels in the amygdala‐piriform region. In the thalamus, the neonatal STZ treatment did not significantly affect the LP level (*p* = 0.46), but SE significantly increased it (*p* = 0.02), although no interaction was found between both factors (*p* = 0.78) (Figure 3).

**FIGURE 4:**
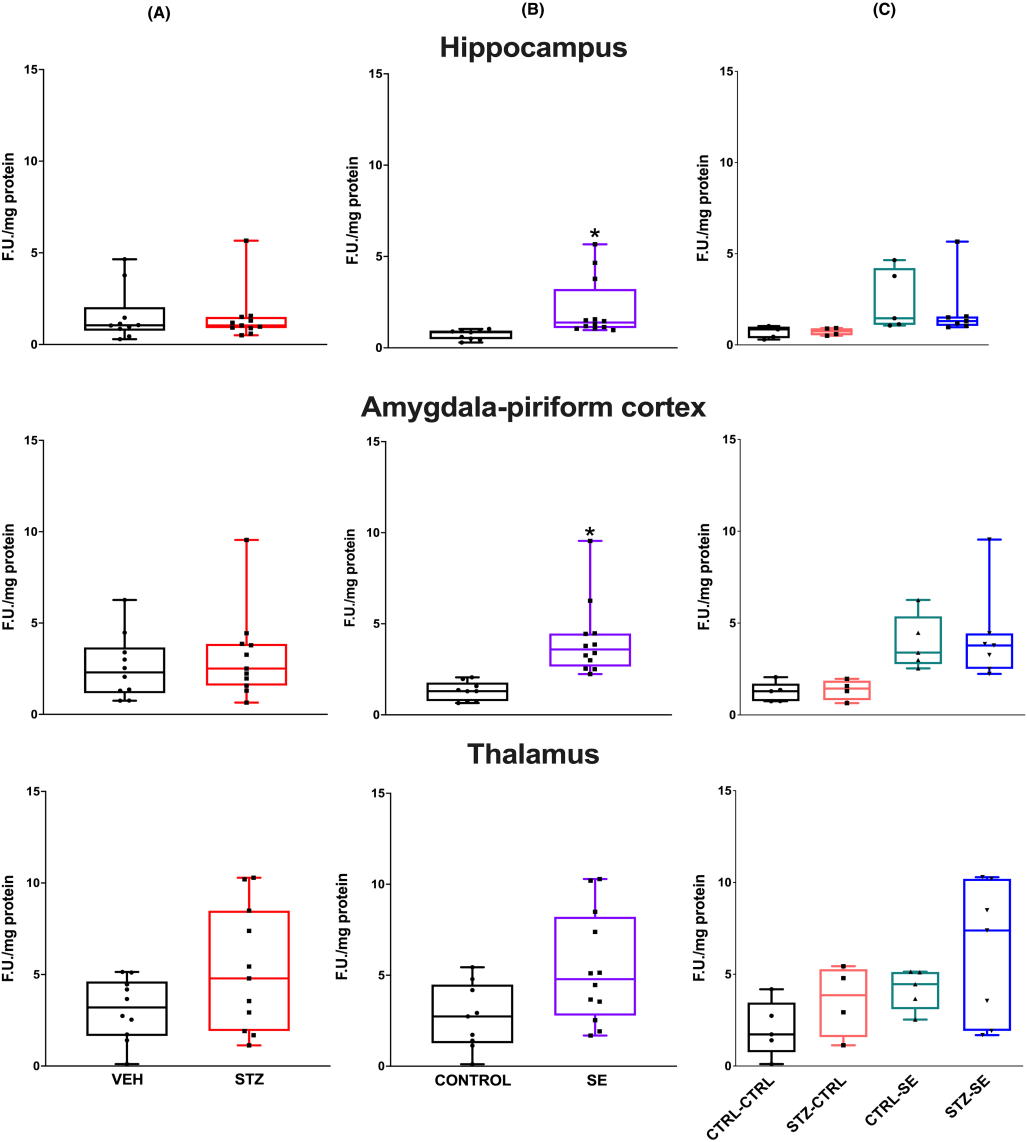
Lipid peroxidation (LP) levels 24 h after SE in the hippocampus, the amygdala–piriform regions, and the thalamus in the different experimental groups. Figures show (A) the effect of neonatal treatment (vehicle, VEH or streptozocin, STZ), (B) the effect of *status epilepticus* (SE vs no seizures, CTRL), and (C) their interaction. Data were analyzed using a two‐way ANOVA and are expressed as the median and interquartile range, the whiskers correspond to the minimum and maximum values for each group. Individual values for each rat are plotted in the graphs. CTRL‐CTRL *n* = 5, STZ‐CTRL *n* = 4, CTRL‐SE *n* = 5 STZ‐SE *n* = 7. *p < 0.05.

When LP was evaluated 24 h after SE onset, the ANOVA analysis showed that the SE induction (*p* = 0.02) was a significant factor in the hippocampus, but neither the neonatal STZ treatment (*p* = 0.66), nor the interaction between both conditions (*p* = 0.64) significantly affected LP levels. A similar phenomenon was observed in the amygdala‐piriform region and an alike tendency in the thalamus, where an increase in LP was observed following SE (*p* = 0.001 and *p* = 0.05, respectively), but neither the neonatal STZ treatment (*p* = 0.77 and *p* = 0.13, respectively), nor the interaction between both conditions (*p* = 0.90 and *p* = 0.83, respectively) were significant factors (Figure 4).

#### 3.4.2 Reactive oxygen species

**FIGURE 5:**
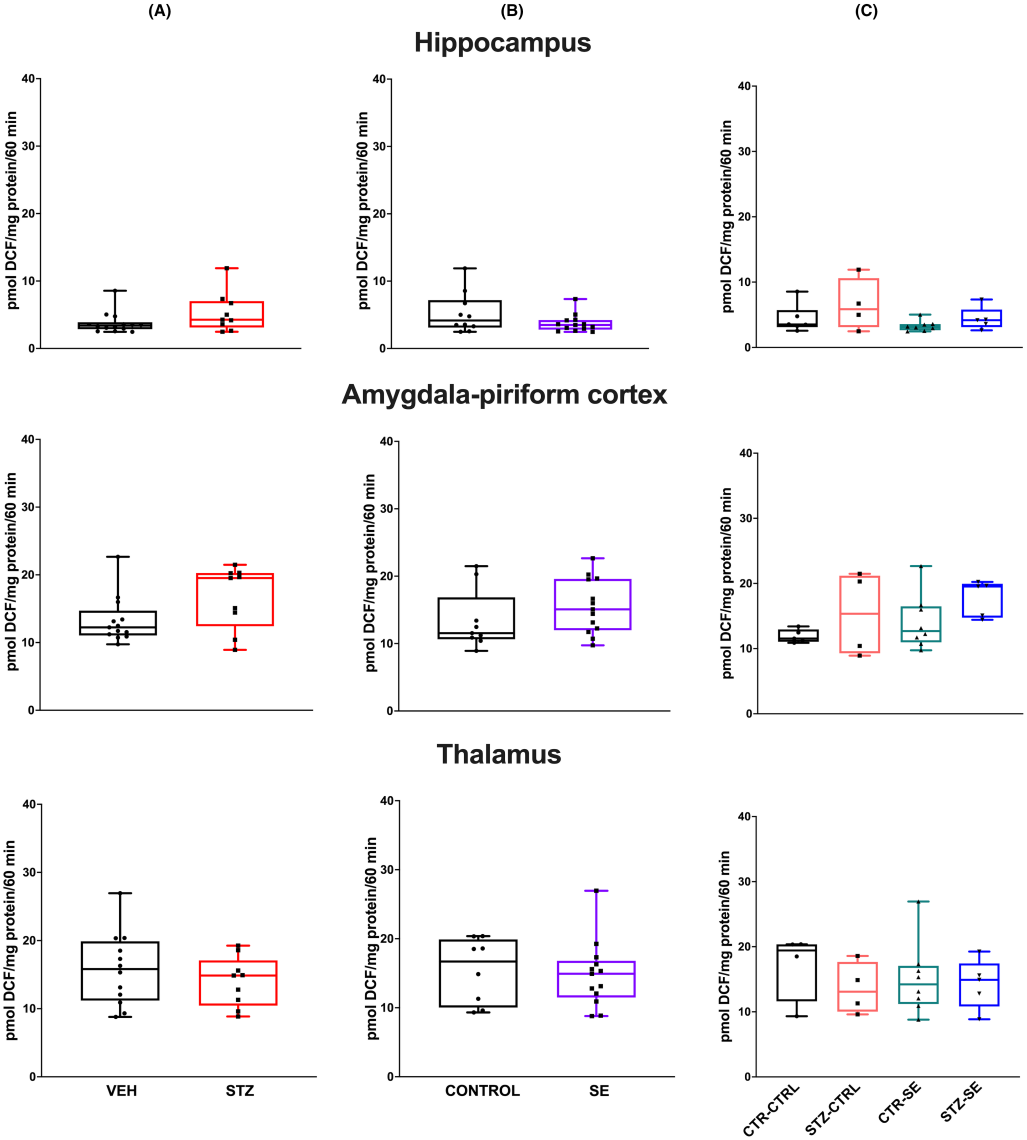
Levels of Reactive Oxygen Species (ROS) 6 h after SE in the hippocampus, the amygdala–piriform regions, and the thalamus in the different experimental groups. Figures show (A) the effect of neonatal treatment (vehicle, VEH or streptozocin, STZ), (B) the effect of *status epilepticus* (SE vs no seizures, CTRL) and (C) their interaction. Data were analyzed using a two‐way ANOVA and are expressed as the median and interquartile range, the whiskers correspond to the minimum and maximum values for each group. Individual values for each rat are plotted in the graphs. CTRL‐CTRL *n* = 5, STZ‐CTRL *n* = 4, CTRL‐SE *n* = 5 STZ‐SE *n* = 7.

ROS levels 6 h after SE and in the control condition were analyzed by two‐way ANOVA analysis. In the hippocampus, neither the STZ neonatal treatment (*p* = 0.94), nor the presence of SE (*p* = 0.10), nor the interaction between both factors (*p* = 0.57) were significant factors. A similar phenomenon was observed in the amygdala‐piriform, where neither the neonatal STZ treatment (*p* = 0.06), nor the SE induction (*p* = 0.19), nor the interaction between both factors (*p* = 0.93) significantly influenced the ROS levels. No differences in thalamic ROS were seen with the neonatal STZ treatment (*p* = 0.34), the SE induction (*p* = 0.76), or their interaction (*p* = 0.54) (Figure 5).

**FIGURE 6:**
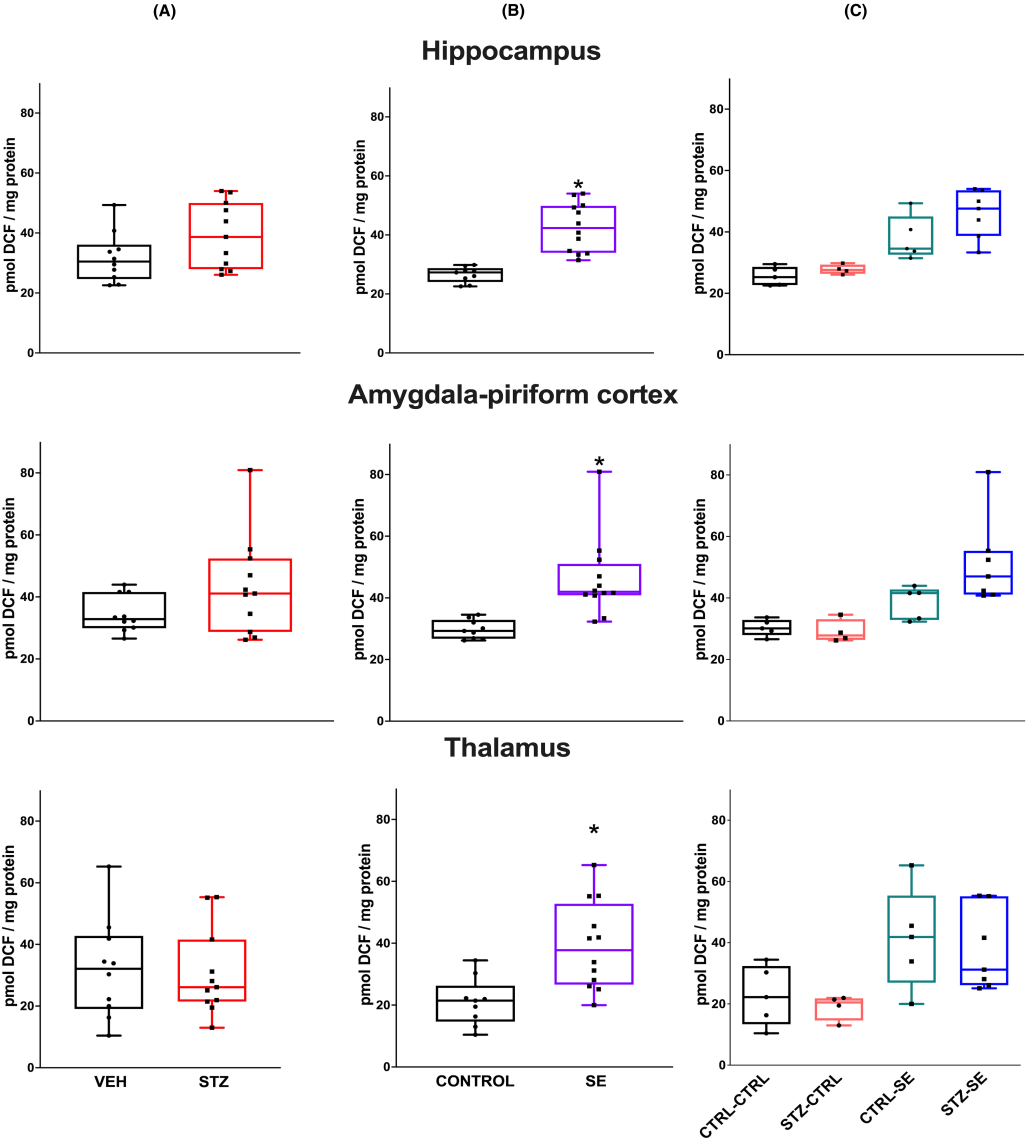
Levels of reactive oxygen species (ROS) 24 h after SE in the hippocampus, the amygdala–piriform regions, and the thalamus in the different experimental groups. Figures show (A) the effect of neonatal treatment (vehicle, VEH or streptozocin, STZ), (B) the effect of *status epilepticus* (SE vs no seizures, CTRL), and (C) their interaction. Data were analyzed using a two‐way analysis of variance (ANOVA) and are expressed as the median and interquartile range. Individual values for each rat are plotted in the graphs. CTRL‐CTRL *n* = 5, STZ‐CTRL *n* = 4, CTRL‐SE *n* = 5 STZ‐SE *n* = 7. *p < 0.05.

SE induction significantly increased the ROS levels in the hippocampus 24 h after SE (*p* < 0.001), but neither the application of neonatal STZ (*p* = 0.75), nor the interaction between both conditions (*p* = 0.30), were significant factors. In the amygdala–piriform, SE significantly increased the ROS levels (*p* = 0.001), but neither the neonatal STZ application (*p* = 0.16) nor the interaction between both conditions (*p* = 0.09) were significant factors. Finally, SE significantly increased the ROS levels in the thalamus (*p* = 0.003), but neither the administration of neonatal STZ (*p* = 0.50) nor to the interaction between both factors (*p* = 0.99) significantly changed the ROS levels (Figure 6).

#### 3.4.3 Correlation among Glycemia and lipid peroxidation or reactive oxygen species

There was a positive correlation between glycemia and LP levels in the hippocampus (*p* = 0.009) from CTRL and STZ rats exposed to SE. It is relevant to mention that the rats administered with STZ (which presented hyperglycemia) showed higher levels of LP. However, no correlation was found between the number and duration of generalized seizures and the LP or ROS levels in any other analyzed brain region (Table S2).

## 4 DISCUSSION

Our results uniquely demonstrate that DM2 exacerbates SE severity and neurodegeneration without affecting seizure‐induced LP or ROS. These findings suggest that DM2 facilitates brain hyperexcitability that makes easier to have a seizure.

Our results show that neonatal STZ administration induced hallmarks of DM2 in adult rats including chronic hyperglycemia, increased HbA1c, low body weight, and altered blood and urine parameters. These signs and symptoms are similar to those observed in people with DM and diabetic rats.^13^, ^14^, ^15^, ^21^, ^22^, ^23^

Our results show that STZ rats (DM2 rats) had increased latency to first generalized seizure and SE onset, along with more severe seizures compared to controls when SE was induced. These results contrast with those reported by Huang and collegues,^8^ who described that rats with DM1 had decreased latency to the first generalized seizure and SE. That difference may be due to dissimilarities in the pathophysiology of DM1 and DM2. The delayed seizure onset in DM2 rats could stem from changes in hippocampal ATP‐sensitive potassium channels (K_ATP_). K_ATP_ channels protect against hypoxia‐induced seizures,^24^ but glucose inhibition of these channels increases neuronal excitability.^25^ These channels allow the flow of potassium at low concentrations of intracellular ATP (less than 1 mM) and close when the intracellular ATP levels increases.^25^ DM2 may produce changes in ATP levels due to the alteration of the intracellular metabolism of glucose: Thus, the dysfunction of the K_ATP_ channels (due to ATP deficiency) may contribute to the presentation of increased latencies in STZ‐administered rats; however, this hypothesis has yet to be demonstrated.

Neuronal cell death induced by SE is mostly due to excitotoxic mechanisms that promote necrosis as the main morphological phenotype of neuronal death,^26^ which can be attributed to OS generated by oxidative mediated by NO^−^.^27^ Our results showed that rats with DM2 showed increased neurodegeneration 24 h after SE in the hippocampus, except for the CA2 area. This exception could be explained due to the high concentrations of acetylcholinesterase in the CA2 field.^28^ These findings agree with those reported previously in C57BL/6 mice with DM1 induced by STZ, which showed a decrease in the number of pyramidal cells in the CA3 and CA1 fields and the hilus 7 days after SE induced by kainate.^7^ A similar result was also described for rats with STZ‐induced DM1 hyperglycemia. Those rats had severe epileptic seizures, increased mortality, and neuronal damage in the CA3 area of the hippocampus 24 h after lithium–pilocarpine administration.^8^ In the hippocampus, diabetic hyperglycemia can promote structural and functional changes due to various factors such as alterations in mitochondrial energy metabolism as consequence of increased mitochondrial ROS, alterations in the NF‐κB pathway, variations in the function of the PTEN protein,^29^ modifications in the integrity of the blood–brain barrier, gliosis, microgliosis, increased expression of pro‐inflammatory markers such as TNF‐α, IL‐6 as well as OS.^30^, ^31^, ^32^, ^33^ The chronic elevation of glucose in DM2 can contribute to increased neurodegeneration in the hippocampus when a severe insult like SE occurs.

In addition to hippocampal damage, our results showed increased neurodegeneration in extra‐hippocampal areas in DM2 rats, such as the piriform cortex, the dorsomedial nucleus of the thalamus, and the medial and cortical nuclei of the amygdala. Direct connections between the hippocampus and these regions may facilitate spread of epileptic activity. High glucose concentrations may cause neuronal hyperexcitability and hypersynchrony,^25^ exacerbating neurodegeneration and epileptogenesis.^34^

One possible explanation for the increased neurodegeneration we observed in DM2 rats could be the OS produced by diabetic hyperglycemia, which in turn may promote mitochondria dysfunction, by altering their membrane potential and bioenergetic metabolic capacity. This increase in SE‐induced damage may be triggered by a decrease in the activity of the mitochondrial aconitase enzyme, promoting lipid peroxidation, and leading to apoptotic cell death as described previously.^35^ However, when we analyzed cerebral OS markers, our results showed no increase in LP or ROS 6 h after SE in any of the analyzed brain region. However, we found a SE‐induced increase in LP levels in the thalamus that could explain the rapid kinetics of neuronal cell death observed in the thalamus after SE^36^ in concordance with our results, but not the neurodegeneration observed in the other brain regions. A previous study showed that OS caused by reactive nitrogen species (RNS) modulates cell death of rat hippocampal neurons 6 h after SE onset.^37^ Since the time necessary to produce NO and ONOO^−^ is shorter than for OH^−^, which is synthesized from H_2_O_2_, it consequently takes longer to produce ROS, which has the highest peroxidant capacity in the lipid membrane.^38^ On the other hand, Bellíssimo and collegues^39^ reported that the activity of the superoxide dismutase enzyme is modified up to 24 h after SE‐induced pilocarpine in adult rats. In addition, these authors measured the concentration of hydroperoxides in the hippocampus, which are intermediate compounds produced as a result of lipid peroxidation promoted by ROS and did not find changes 5 h after SE, but they did find differences 24 h after seizures. When we analyzed the indicators of cerebral OS at 24 h, we found that SE increased LP levels in both control and STZ rats, which is consistent with previous research reporting increased malonaldehyde levels and nitrite levels, and decreased activity of antioxidant enzymes in the hippocampus of rats that had SE induced by the lithium–pilocarpine model.^40^ Hyperglycemia is known to promote a neuro‐inflammatory environment, by promoting the activation of microglia and astroglia, which is associated with high levels of brain NO^−^ and OS.^30^ However, our findings indicate SE elicits a greater oxidative damage than DM2 hyperglycemia alone.

There are several mechanisms through which SE promotes the development of OS. Among them, metabolic and mitochondrial alterations stand out, both morphologically and functionally, and can promote and maintain neuronal excitability.^35^ Regarding the brain oxidative damage related to DM, it has been found that hyperglycemia promotes increased levels of NO^−^ and LP both in plasma and in the liver and brain tissue, but also an increase in antioxidant activity of superoxide dismutase (SOD), glutathione peroxidase and glutathione, as well as the levels of proinflammatory molecules such as IL‐6 and TNF‐α as early as 30 days after induction of diabetes DM1 with STZ in rats.^41^ In another study carried out in the hippocampus of adult rats after 8 weeks of STZ application to induce DM1, an increase in the concentration of NO^−^ and malondialdehyde (MDA) levels was reported along with a decrease in the antioxidant activity of MDA and SOD.^42^ This suggests that, although diabetic hyperglycemia establishes OS states in the early stages, compensatory mechanisms are also activated in the brain to counteract the oxidative damage, such as the overactivation of antioxidant defense.^39^ When analyzing the mitochondrial function of rats with early DM1, oxygen consumption increases were found in the mitochondria isolated from the cerebral cortex, hippocampus, and cerebellum, whereas the mitochondrial membrane potential is reduced only in the hippocampus, and the content of ATP, cytochrome c and glutathione levels were similar in the three brain regions from normoglycemic rats. This indicates that the metabolic response to hyperglycemia is different in each brain structure and does not induce metabolic alterations in those structures in the early stages.^43^ Taken together, these data could explain the reason why the OS caused by DM2 under our experimental conditions is not greater than the one caused by SE under normoglycemic conditions. In addition, in a recent study, it was shown that glucose control before and after SE does not interfere with the establishment of brain OS.^44^

A limitation of our study is that we evaluated the early stages of DM2, and brain changes could be less pronounced compared with the advanced stages of the disease. Although we analyzed ROS, it would also be relevant to study RNS and enzymes involved in counteracting OS, which would help us to understand the relationship between DM2 and seizures. Another caveat is that there exists variability in some data points, and future experiments may need a larger sample size.

In conclusion, DM2 exacerbated SE severity and neurodegeneration but did not modify SE‐induced OS under these conditions.

## AUTHOR CONTRIBUTIONS

María Leonor López‐Meraz contributed to the study conceptualization and design, material preparation, and provided study resources. Karen Paola Ramos‐Riera wrote the first draft of the manuscript. Karen Paola Ramos‐Riera, Pablo Eliasib Martínez‐Gopar, and María Leonor López‐Meraz performed the experiments and contributed to data acquisition and analysis. Francisca Pérez‐Severiano, Luis Beltrán‐Parrazal, and Consuelo Morgado‐Valle provided study resources and contributed to data analysis and writing—review and editing. All authors commented on previous versions of the manuscript. All authors read and approved the final manuscript.

## FUNDING INFORMATION

KPRR received a scholarship for postgraduate studies (893823) granted by the National Council of Science and Technology of Mexico (CONAHCyT). The Dirección General de Investigaciones at the Universidad Veracruzana awarded support for the publication of this research to ML‐LM.

## CONFLICT OF INTEREST STATEMENT

None of the authors has any conflict of interest to disclose.

## ETHICAL PUBLICATION STATEMENT

We confirm that we have read the Journal's position on issues involved in ethical publication and affirm that this report is consistent with these guidelines.

This study was carried out in strict accordance with the Guide for the Care and Use of Laboratory Animals of the National Institutes of Health and under the ARRIVE guidelines.

## Supporting information


**Supinfo S1.**.

## Data Availability

Data are available upon reasonable request.
