# Supplementary material for: Type 2 diabetes mellitus facilitates status epilepticus in adult rats: Seizure severity, neurodegeneration, and oxidative stress
Source: Epilepsia Open. 2024 Feb 6;9(2):665–78. doi: 10.1002/epi4.12905 (PMC10984310; doi:10.1002/epi4.12905)
Supplement: Supplementary file 1 — Supinfo S1.. [file EPI4-9-665-s001.docx]

**Supplementary materials**

**Type 2 diabetes mellitus facilitates *status epilepticus* in adult rats: seizure severity, neurodegeneration, and oxidative stress.**

Ramos-Riera Karen Paola, Beltrán-Parrazal Luis, Consuelo Morgado-valle, Pérez-Severiano Francisca, Martínez-Gopar Pablo Eliasib, López-Meraz María Leonor

**Methods**

**Determination of biochemical indicators of diabetes**

A group of rats neonatally administered with STZ or vehicle (CTRL group) were euthanized by decapitation at P90, their brain was used for OS determination as described below, and 2-3 mL of blood from the carotid artery was collected in tubes with or without anticoagulant (EDTA) to evaluate their metabolic status. Blood samples were stored at 4 °C until analysis. Serum was obtained immediately after clot retraction and centrifugation at 3000 rpm for 10 min and stored at -18 °C until analysis.

a) Glycated Hemoglobin. The percentage of glycated hemoglobin A1c was quantitatively determined *in vitro* in whole blood by automated spectrophotometry with the ARCHITECT c 4000 and c 8000 analyzers.

b) Urea and blood urea nitrogen. Urea levels were measured by endpoint enzyme assay in whole blood following the manufacturer`s instructions (Urea Nitrogen kit Ref. 0018255440 quantILAB, Instrumentation Laboratory, Werfen Company). Results for urea levels are reported in mg/dL. To calculate the blood urea nitrogen (BUN) the urea levels were divided by 2,145 and are shown in mg/dL.

c) Creatinine. Determination was performed by endpoint enzymatic analysis in whole blood following the manufacturer`s instructions (Creatinine Enzymatic kit Ref 0018259440 quantILAB, Instrumentation Laboratory, Werfen Company). Results are reported in mg/dL.

Urinary biochemical indicators of diabetes were also analyzed. For this purpose, urine from CTRL and STZ rats was collected for 1 h previous to euthanasia; access to water and food was restricted during this period. We determined protein, glucose, ketone bodies, and specific gravity by using urinalysis reagent strip pads (Mission). Once the urine was obtained, strip pads were impregnated with the samples, and the results were compared with the color chart found on the tube labeling for each parameter. The results were obtained by direct comparison with the color chart.

**Induction of *status epilepticus***

At P89 rats were i.p injected with lithium chloride (3 mEq/kg equivalent to # L4408 Sigma), and 18 h later, SE was induced by subcutaneous injection of pilocarpine hydrochloride (30 mg/Kg # P6503 Sigma) (Jope et al., 1986). The rats were individually placed in acrylic boxes, and behavioral seizures were video-recorded and analyzed offline. Seizure severity was evaluated with the behavioral scale described by Racine (1972): stage I= facial clonus; stage II= stage 1 behavior and nodding head; stage III= stage 2 behaviors and forelimbs clonus; phase IV= stage 3 behavior and rearing; phase V = stage IV behaviors loss of postural tone. SE was defined as continuous seizure activity for at least 30 minutes. One hour after SE onset, rats received an injection of 10 mg/Kg i.p. of diazepam (Relazepam Vet, PiSA) to stop SE and to increase survival. Subsequently, rats were housed individually in an acrylic box with water and food ad libitum.

**Determination of neurodegeneration**

**Transcardiac perfusion and tissue processing**

Twenty-four hours after SE, a group of rats were anesthetized with 120 mg/Kg, i.p. sodium pentobarbital (Cheminova), and under deep anesthesia were placed in a supine position to expose the heart. Then 250 mL of saline solution (SS; 0.9%) was transcardially perfused at 20 mL/min flow, followed by 250 mL of 4% paraformaldehyde (dissolved in a 0.1 M phosphate solution, pH 7.4). The brains were kept in situ overnight at 4°C, removed, and additionally post-fixed in 4% paraformaldehyde solution for 2 h. After that time, the tissue was dehydrated using ethanol solutions at increasing concentrations (70, 80, 95, and 100%) and xylol. At the end of this process, the brain tissue was immersed in paraffin (Paraplast Plus, Sigma P3683) and finally embedded in paraffin blocks. Serial 10 μm coronal sections were obtained at the level of the dorsal hippocampus (3.36 mm posterior to bregma, Paxinos, and Watson 2007) using a Leica microtome.

**Fluoro-Jade B (F-JB) stain**

We assessed neurodegeneration by using the F-JB staining. Brain sections were deparaffinized and incubated in 0.06% potassium permanganate (KMnO4) for 30 min. Subsequently, the sections were washed with distilled water and placed in a 0.001% F-JB (Chemicon International AG310) for 30 minutes in the darkness. After this time, sections were washed with water to remove the excess of the dye and left to dry at room temperature and protected from light. Twenty-four hours later, the slides were dehydrated in 95% and 100% ethanol, rinsed with xylene for 2 min, and mounted with a non-aqueous medium for subsequent analysis under the Olympus AX70 microscope (U-MWB filter at an excitation of 450-480 nm). The brain regions evaluated were the granular layer of the dentate gyrus (DG), the hilus, CA3, CA2, and CA1 pyramidal layer of the hippocampus, layer II of the piriform cortex, the dorsomedial nucleus of the thalamus (which includes the medial dorsal medial, central and lateral nuclei), the lateral amygdala (including the lateral dorsolateral, lateral ventromedial, and lateral ventrolateral nuclei), the medial amygdala (including the medial posterodorsal nucleus and medial posteroventral nucleus), and the cortical amygdala (including the posteromedial cortical nucleus and area amygdala anterolateral hippocampus). Positive F-JB cells were analyzed in 40X and 10x magnification photomicrographs acquired with the Leica Application System LAS V 4.8 software. The number of positive F-JB cells was determined with the Fiji ImageJ program for Windows. Four consecutive brain slices were analyzed unilaterally for each rat, randomly selecting the right or left hemisphere. The counts are reported as the number of cells/mm2 for each brain region evaluated.

**Oxidative stress analysis**

To evaluate the OS, we measured the products of lipid peroxidation (LP) and the formation of ROS 6 and 24 h after SE. The rats were decapitated, and the brain regions of interest (hippocampus, amygdala-piriform cortex region, frontal cortex, and thalamus) were quickly dissected on ice. The tissues were kept deep-frozen at -80 °C until analysis.

**Lipid peroxidation**

The tissue was homogenized in 2 mL of SS, and then, two aliquots of 900 µL were separated and added to 4 mL of a chloroform-methanol solution (2:1 vol/vol) in tubes protected from light. The tubes were kept under vortex agitation for 10 s, and then the samples were incubated for 30 min at 4°C and in the dark to allow phase separation. The upper phase (methanol) was removed using a vacuum system, and 2 mL of the organic phase (chloroform) were transferred to a quartz cell adding 200 µL of methanol and wad with a fluorescence spectrophotometer (Perkin Elmer LS50B) using 370 nm and 430 nm excitation and emission wavelengths, respectively. A quinine solution (0.001 mg/mL) was used as a reference. Fluorescence values were corrected with the corresponding protein content. Results are expressed as F.U./mg protein (Pérez-Severiano et al., 2013).

**Reactive Oxygen Species**

The formation of ROS was determined using 2'7'- dichlorodihydrofluorescein diacetate (DCFH- DA). A dilution (1:10) of the sample homogenate was prepared with SS. Subsequently, 5 µL aliquots were incubated with 145 µL of TRIS- HEPES buffer (18:1) and 50 µL of DCF-DA solution (50 µM) in a 96-well microplate at 37°C for 1 h in agitation. The fluorescence of each sample was determined on an FLx800 multi-plate reader (Biotek Instruments, Inc.), using 488 nm and 525 nm as excitation and emission wavelengths, respectively. Results were calculated from a calibration curve of DCF and adjusted according to the protein content. Results are expressed as pmol DCF/mg protein/60 min (Martínez-Gopar et al., 2022).

**Figures**


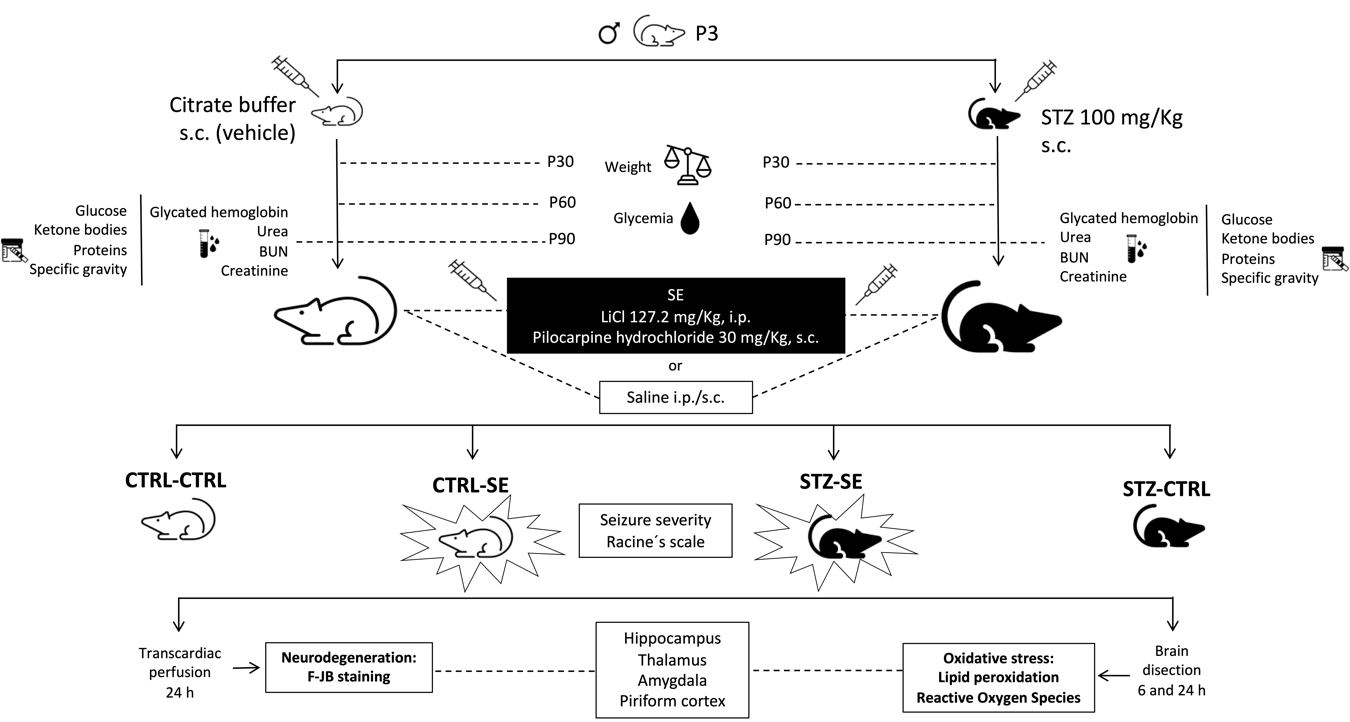


Figure S1. The diagram represents the general methodology followed for this study. Diabetes mellitus was induced by streptozocin (STZ) on postnatal day 3. Vehicle was administered as a control (CTRL). Blood glucose and weight measurements were performed at postnatal day (P) 30, 60 and 90; at P90 biochemical indicators of diabetes were analyzed in blood and urine. Status epilepticus (SE) was induced using the lithium-pilocarpine model in CTRL and STZ groups (CTRL- SE and STZ-SE groups); CTRL and STZ groups without seizures were injected with LiCl and saline (CTRL-CTRL and STZ-CTRL). Twenty-four hours after the start of SE or the control condition, subjects on the 4 experimental groups underwent transcardiac perfusion and the brain was obtained, paraffin-fixed, and analyzed for neurodegeneration by staining with Fluoro-jade B (F-JB). Other rats from the 4 groups experimental groups were decapitated 6 or 24 hours after the start of SE, or the control condition, for the determination of cerebral oxidative stress by measuring lipid peroxidation (LP) and reactive oxygen species (ROS).


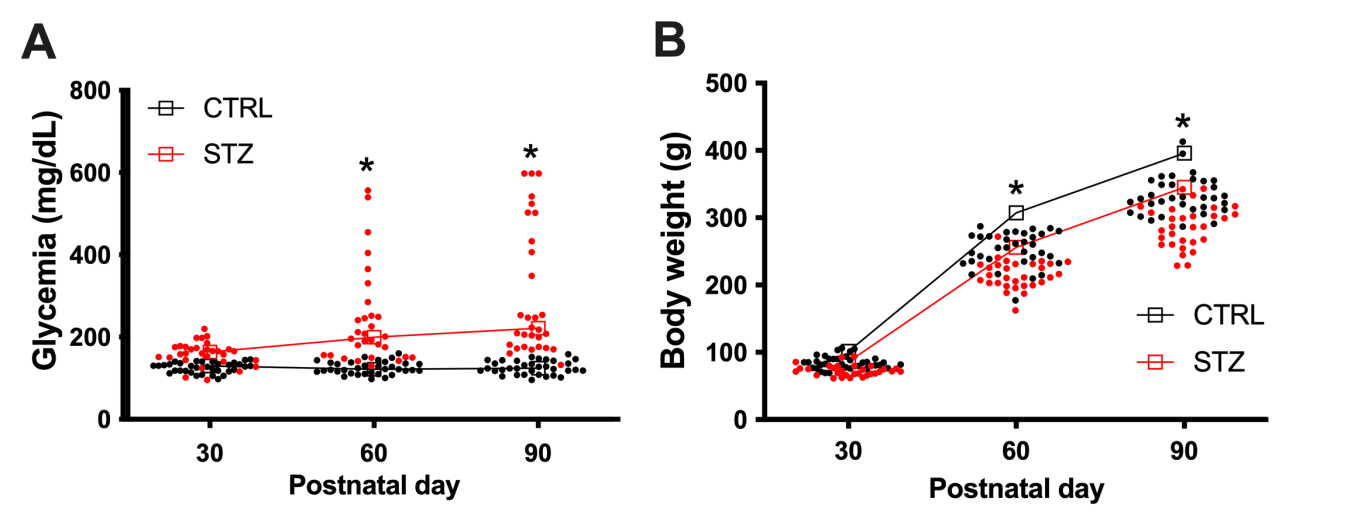


Figure S2. Metabolic changes induced by neonatal application of streptozocin (STZ) are similar to those observed in diabetic mellitus type 2. (A) Time course of glycemia and (B) body weight of rats administered with STZ or vehicle (CTRL) during the postnatal day 3. Evaluation was performed on postnatal days 30, 60 and 90. Data are expressed as mean S.E.M. Individual values for each rat are plotted in the graphs. CTRL Group n=34, STZ Group n=30. **p*<0.0001.

**Tables**

Table S1. Biochemical parameters of diabetes mellitus in blood and urine of neonatally administered adult rats with vehicle (CTRL) or streptozocin (STZ).

| **Experimental group** | **Biochemical parameters**  **in blood** | | | | **Biochemical parameters in urine** | |
| --- | --- | --- | --- | --- | --- | --- |
|  | **HbA1c**  **(%)** | **Urea**  **(mg/dL)** | **Ureic nitrogen**  **(mg/dL)** | **Creatinine**  **(mg/dL)** | **Specific gravity** | **Proteinuria**  **(mg/dL)** |
| CTRL  (n=9) | 3.7  (3.4/3.9) | 20.7  (18.2/38) | 14.8  (8.5/35.5) | 0.2  (0.2/0.6) | 1.01  (1.01/1.01) | 0  (0/0) |
| STZ  (n=10) | 4.4*  (4.1/6.4) | 46.35*  (19.7/230.2) | 21.6*  (9.2/107.9) | 0.4*  (0.3/0.9) | 1.015*  (1.01/1.02) | 15.0*  (0/100) |

The results are expressed as the median and the minimal/maximal value (in parentheses) and were analyzed with the U-Mann-Whitney (UWM) test to evaluate differences between groups. **p*<0.05.

Table S2. Correlation between blood glucose levels, number and duration of generalized seizures, and lipid peroxidation (LP) or Reactive Oxygen Species (ROS) production in different brain regions of rats.

| **Brain region/ Oxidative stress marker** | **Glycemia** | | | **Frequency of generalized seizures** | | | **Duration of generalized seizures** | | |
| --- | --- | --- | --- | --- | --- | --- | --- | --- | --- |
|  | **r** | **r^2^** | **p** | **r** | **r^2^** | **p** | **r** | **r^2^** | **p** |
| **Lipid peroxidation** | | | | | | | | | |
| Hippocampus | 0.6880 | 0.4734 | 0.009* | 0.04106 | 0.0016 | 0.89 | 0.3721 | 0.1385 | 0.21 |
| Amygdala- Piriform cortex | -0.2903 | 0.0842 | 0.08 | -0.0937 | 0.0087 | 0.76 | 0.07163 | 0.0051 | 0.81 |
| Thalamus | 0.3010 | 0.0906 | 0.31 | 0.4400 | 0.1936 | 0.13 | 0.1288 | 0.0165 | 0.67 |
| **ROS** | | | | | | | | | |
| Hippocampus | 0.0721 | 0.0052 | 0.81 | 0.3300 | 0.1089 | 0.27 | 0.2689 | 0.0723 | 0.37 |
| Amygdala- Piriform cortex | 0.0712 | 0.0050 | 0.81 | 0.1821 | 0.0331 | 0.55 | 0.2256 | 0.0509 | 0.45 |
| Thalamus | -0.1539 | 0.0237 | 0.61 | 0.2184 | 0.0476 | 0.47 | -0.1018 | 0.0103 | 0.74 |

Generalized seizures considered stage IV and V seizures. Data were analyzed with Pearson's correlation coefficient. Table shows the Pearson the correlation coefficient (r), the determination coefficient (r^2^) and the *p* value. **p*<0.05.
